# Closed-Loop tACS Delivered during Slow-Wave Sleep Reduces Retroactive Interference on a Paired-Associates Learning Task

**DOI:** 10.3390/brainsci13030468

**Published:** 2023-03-09

**Authors:** Aaron P. Jones, Natalie B. Bryant, Bradley M. Robert, Teagan S. Mullins, Michael C. S. Trumbo, Nicholas A. Ketz, Michael D. Howard, Praveen K. Pilly, Vincent P. Clark

**Affiliations:** 1 Psychology Clinical Neuroscience Center, Department of Psychology, The University of New Mexico, Albuquerque, NM 87131, USA; 2 Department of Psychology, The University of New Mexico, Albuquerque, NM 87131, USA; 3 Proficient Autonomy Center, Intelligent Systems Laboratory, HRL Laboratories, LLC, Malibu, CA 90265, USA; 4 The Mind Research Network, Albuquerque, NM 87106, USA; 5 Department of Neuroscience, University of New Mexico School of Medicine, Albuquerque, NM 87106, USA

**Keywords:** closed-loop transcranial alternating current stimulation (CL-tACS), sleep-dependent memory consolidation, retroactive memory interference, slow-wave sleep, learning and memory, cognitive enhancement

## Abstract

Previous studies have found a benefit of closed-loop transcranial alternating current stimulation (CL-tACS) matched to ongoing slow-wave oscillations (SWO) during sleep on memory consolidation for words in a paired associates task (PAT). Here, we examined the effects of CL-tACS in a retroactive interference PAT (ri-PAT) paradigm, where additional stimuli were presented to increase interference and reduce memory performance. Thirty-one participants were tested on a PAT before sleep, and CL-tACS was applied over the right and left DLPFC (F3 and F4) vs. mastoids for five cycles after detection of the onset of each discrete event of SWO during sleep. Participants were awoken the following morning, learned a new PAT list, and then were tested on the original list. There was a significant effect of stimulation condition (*p* = 0.04297; Cohen’s *d* = 0.768), where verum stimulation resulted in reduced retroactive interference compared with sham and a significant interaction of encoding strength and stimulation condition (*p* = 0.03591). Planned simple effects testing within levels of encoding revealed a significant effect of stimulation only for low-encoders (*p* = 0.0066; Cohen’s *d* = 1.075) but not high-encoders. We demonstrate here for the first time that CL-tACS during sleep can enhance the protective benefits on retroactive interference in participants who have lower encoding aptitude.

## 1. Introduction

Humans spend roughly one-third of their lives asleep [1]. Sleep is a universal phenomenon and has been identified in every animal studied [2,3]. It is a homeostatic process, governed by a circadian system [4], disruption of which leads to cognitive inefficiency [5]. Sleep is characterized by two main stages: rapid eye movement (REM) and non-REM (NREM) sleep. These stages are discernable by collecting electroencephalography (EEG) data during sleep, a method called polysomnography. Slow-wave sleep (SWS), the deepest phase of NREM, is rich in the first half of the night, decreasing in distribution and density throughout the night, and is marked by slow high-amplitude oscillations, called slow-wave activity (SWA), which peaks at around 0.75 Hz [6]. REM sleep, in contrast, is characterized by low-amplitude fast oscillatory brain activity that resembles waking EEG and muscle atonia. Sleep is crucial for the consolidation of memory, though the contribution of different sleep stages to domain-specific memory consolidation [7,8,9], and how memories change from waking to sleep [10] is unclear. SWS appears to be involved in declarative memory consolidation [8,9,11,12], while REM sleep is thought to be involved in procedural memory consolidation [8].

### 1.1. Sleep and Memory Interference

The author of [13] suggests that sleep may play a role in reinforcing and protecting knowledge. SWS is thought to be an important factor in the protective benefit of sleep on retroactive interference and on long-term memory formation [14], and this phenomenon has been studied for decades [15].

Interference paradigms are distinguished by whether the words in each list are unique. In the so-called AB-AC paradigm, also known as associative interference, the “B” word in list AB is replaced with “C” words in the AC list. In the so-called AB-CD paradigm, also known as non-associative interference, each word-pair list is unique. Both paradigms can result in previously learned information inhibiting learning of new information (proactive interference), or in newly learned information hindering previously learned information (retroactive interference) [16]. In [16], participants performed a paired associates task (PAT) in a series of experiments utilizing either associative or non-associative interference paradigms after participants either slept or stayed awake after learning. The results suggested that sleep following learning results in less interference, but no difference was found between types of interference. Sleep also specifically affected weakly encoded memories from retroactive interference. The authors of [17] had participants perform a verbal PAT in an interference paradigm. They first learned list AB, followed by 12 h of sleep or wakefulness. Each group was then split into interference groups, where one group learned a new list (AC) in an associative interference AB-AC paradigm, while the other group did not. They were then tested on list AB. Learning criteria for both lists were set at 100%. The results suggested that the sleep group overall had slightly better memory performance compared to the wake group in the no interference condition. However, in the interference condition, a highly significant difference was found between the sleep and wake groups, where the sleep group had much better memory performance. In [18], a modified AB-AC interference PAT was used, where half of the listed AB words were paired with items in the AC list, while the rest were unique. Participants learned list AB, and then either took a nap or stayed awake, then learned list AC, and, finally, were tested on list AB in a within-subjects design separated by one month. The results suggested that sleep, specifically naps with SWS, protected information from retroactive interference. They concluded that SWS, but not REM sleep, is important for protection from post-sleep retroactive interference.

Encoding strength during learning appears to affect sleep-dependent memory as well. In [19], participants performed a verbal PAT with interference in four groups: a sleep group, a wake group, an immediate recall group, and a delayed recall group. In a second experiment, the strength of encoding was manipulated in learning the lists in a non-associative AB-CD interference paradigm. In the intense encoding condition, feedback was displayed for longer, and participants had to reach 90% performance. In the weak encoding condition, feedback was shorter, where 60% performance was required. Results from the first experiment suggested that after sleep list AB performance was better than in the waking condition. However, list AC showed no effect of sleep. Furthermore, there was no effect of immediate or delayed recall. The results of experiment two suggested that strongly encoded word pairs were better remembered; however, there was an effect on weakly encoded information only in the sleep group, suggesting that sleep preferentially benefits weakly encoded information. It is important to note that participants recalled fewer words at the test than encoding in all conditions. The authors suggested that sleep is associated with declarative memory consolidation and may preferentially benefit weakly encoded information. Further evidence of encoding strength effects comes from [20], where participants performed a PAT in an AB-AC interference paradigm. Participants either slept or stayed awake following AB list learning. A second experiment had participants take a nap instead of sleeping a full night. Participants were trained to 100% correct recall on lists AB and AC. Results suggested no effect of sleep (either nap or full night) on interference. In fact, retroactive interference was only seen after sleep but not after wake. They suggested that subjects rapidly encoded words into the neocortex (criterion set to 100%), thus sleep consolidation was not necessary, and only weakly encoded information would benefit from sleep-dependent memory consolidation processes.

### 1.2. Electrical Augmentation of Memory

There are a variety of ways to augment consolidation in humans, including pharmacologically [21], by utilizing the testing effect [22], distributed learning [23], dreaming [24], and manipulation of physiological states [25], among others. Recently, transcranial current stimulation (tCS), including transcranial direct and alternating current stimulation (tDCS, tACS, respectively), has become a popular method to communicate with the brain in its own language, electricity [26], as well as to augment cognition [27]. Utilizing transcranial current stimulation (tCS) to enhance cognitive functioning is becoming increasingly popular [28]. tCS may be involved in neural plasticity [29], and several review papers and meta-analyses have shown the efficacy of tCS in enhancing cognitive performance in both healthy and clinical populations [27,30,31,32]. There is considerable evidence that transcranial alternating current stimulation (tACS) is effective in entraining brain oscillations [33,34,35,36]. Whereas tDCS affects the firing rate, tACS regulates the firing rate in an oscillatory manner without affecting the average overall rate, and even low-amplitude AC stimulation results in increased coherence between neuronal spikes and frequency of stimulation. In [35,36], they suggested that tACS is a promising tool to modulate brain areas relevant to distinct cognitive functions to determine their functional impact. tDCS can be applied in different waveforms in a constant or oscillatory manner (e.g., square, trapezoidal, sinusoidal), with resulting entrainment effects for transcranial oscillatory direct current stimulation (toDCS). The difference is that tDCS does not change polarity (i.e., is either positive or negative), whereas tACS rhythmically shifts polarity, which influences the depolarization and hyperpolarization states of the affected neurons. The studies of [33,37] suggested that the phase at which tACS is applied is crucial. Closed-loop transcranial alternating current stimulation (CL-tACS), a technique that involves using feedback from the brain to inform stimulation parameters without online human intervention, provides the most optimized method for delivering stimulation and entraining neurons in the human brain and has only recently been demonstrated [38]. Being able to detect online changes to endogenous brain rhythms and apply targeted stimulation is a promising method by which to invoke change in brain function and behavior modulation.

### 1.3. Electrical Augmentation of Memory during Sleep

Augmentation of memory consolidation during sleep has previously been explored using pharmacological methods [39,40], auditory stimulation [41], Targeted Memory Reactivation (TMR; [42,43,44,45]), and even by physically rocking participants during sleep [46]. The use of tDCS or tACS during sleep has been shown to improve memory performance in various ways, including recall [6,47] and encoding [48]. It could be a rehabilitation tool for those afflicted with sleep disorders, such as chronic insomnia [49], epilepsy [50,51,52], or for children with attention deficit hyperactivity disorder (ADHD, [53]). In a recent meta-analysis, [54] provided evidence that electrical stimulation during sleep improves declarative memory, while leaving procedural memory unchanged, with an average effect size (SMD) of 0.447 for declarative memory enhancement, and −0.476 for disruption, both of which are significant. The enhancement of SWS appears to be particularly important for the effects, where these procedures drive the brain from electrodes placed on the scalp, mainly over the bilateral dorsolateral prefrontal cortex (DLPFC; [6,47]) or by using transcranial magnetic stimulation (TMS; [55]), leading to an increase in nearly global SWA, whereas median nerve stimulation during SWS led to an increase in SWA that was restricted to somatosensory and motor cortices [2], suggesting targeting of specific brain regions is possible if necessary.

### 1.4. Slow-Wave Sleep

The authors of [6] administered 0.26 mA/cm^2^ anodal tDCS over bilateral fronto-cortical electrode sites during wake and separately during SWS-rich sleep stages repeatedly for 30 min in 15 s on/off cycles. The results suggested that declarative memory retention was superior in the active group only when stimulation was delivered during sleep compared to a placebo stimulation group; there was no effect for the waking cohort. The authors of [47] used toDCS during SWS. A 0.75 Hz (maximum current density: 0.517 mA/cm^2^) trapezoidal wave was applied over bilateral frontal cortex in 5, 5 min intervals after subjects had entered SWS. The enhancement of SWS with tACS led only to an improvement in the verbal PAT; however, no benefit was observed for the non-verbal PAT, suggesting that this intervention is specific to verbal memory. To dissociate the effect of slow oscillation enhancement on learning, 5 Hz theta stimulation was applied during SWS. Compared with sham stimulation, theta stimulation reduced slow oscillation power and frontal slow-spindle power and did not benefit word-pair learning. In a follow-up study investigating the effect of theta-band stimulation over DLPFC during either stable NREM sleep or during REM sleep, [56] found that 5 Hz sinusoidal toDCS during NREM produced an impairment in declarative memory consolidation, assessed with a PAT, while leaving performance on procedural tasks unchanged. The authors of [57,58] aimed to use tES to strengthen endogenous slow-wave oscillations (SWOs) and enhance memory consolidation. Their target detection task involved learning to identify threats present in an image through a discovery learning paradigm, and the tests involved correctly distinguishing images in which there was a hidden threat present versus those where threats were absent. They applied 1.0 mA of tDCS during training, and 1.5 mA per hemisphere of CL-tACS over the right and left DLPFC to active subjects during slow wave up states, where the phase and frequency was matched to the endogenous oscillations. Sham subjects received 0.1 mA tDCS over the right DLPFC during training and no stimulation during sleep. The authors of [58] found that active closed-loop tACS was associated with an increase in slow wave power to spindle coupling as well as an improved accuracy with stimulation. The authors of [57] found that there was a stimulation dose-dependent enhancement of memory, such that memory was boosted the most for the subjects who received a number of stimulation events during the night that was closest to the median. This paradigm also resulted in improved subjective sleep quality of participants [59].

This effect is apparent even after a 90 min nap. The authors of [60] delivered 0.25 mA or sham toDCS during two naps in 15 participants, separated by a month, after a learning phase in several cognitive tasks, including a PAT. The time spent in SWS, fast and slow spindle power, and an improvement in recall in the PAT were observed in the active stimulation conditions, compared to the sham. The authors of [61] delivered toDCS on older adults during SWS in a 90 min nap. Bilateral 0.75 Hz sinusoidal toDCS (0.0–260 µA) was administered over lateral frontal cortices for the active group, and no stimulation was administered for the sham group. The results suggested that SWO amplitude was higher in the experimental group, and this group showed a larger improvement in word-pair recall compared to the sham group. Results in this sample are inconsistent [62], but incongruent experimental procedures may be responsible. These findings have not always been replicated [63]. For example, [64] failed to show a benefit of toDCS on declarative memory, assessed via a PAT, in 26 elderly adults. Subjects spent less time in SWS and more overall time awake in the stimulation conditions, compared to the sham. Another study, [65], also failed to find an effect in an older, healthy sample, however, the position of the electrodes was different than previous studies (electrode locations Fp1/Fp2 and P3/P4). The authors of [66] also showed that SWA and spindle activity was enhanced with SWS stimulation in 21 healthy older adults, but they failed to produce a benefit in a PAT. Additionally, tACS during sleep may not always show a neural entrainment effect. For example, [67] found no entrainment effect of low amplitude tACS (2.5 mA) on slow oscillations during SWS using implanted recording electrodes in epileptic patients. For an excellent review on this body of work, see [68].

### 1.5. Aims and Hypotheses

Sleep is universal and understanding it more fully could lead to a host of benefits, from improved cognitive performance to improved quality of life. Memory consolidation during wake and sleep is a complicated process, and there are many unanswered questions. tCS is a tool that can be used to both help us to understand, as well as augment, sleep-dependent memory consolidation processes, as well as the quality of sleep in general. The current study was designed to investigate whether CL-tACS delivered during SWS can prevent retroactive interference in a declarative memory task using a between-subjects design comparing verum stimulation to sham (no stimulation), modeled after [6,47].

The experimental questions were: (1) Does active CL-tACS protect from retroactive interference of list C-D (learned in the morning following sleep) on list A-B (learned in the evening prior to sleep) recall? (2) Does higher initial encoding strength of list A-B and stimulation interact to influence performance? Hypotheses for this experiment were: (1) Verum stimulation will lead to less interference of list C-D learning on list A-B test performance. (2) Low encoders on list A-B will show a larger benefit of stimulation compared to high encoders.

## 2. Materials and Methods

### 2.1. Participants

Participants were men and women who were between 18–40 years of age (mean age = 20.41, SD = 1.701), used English as a first language, completed high school, and had no history of head injury with loss of consciousness for longer than five minutes. They were right handed according to the Edinburgh Handedness Inventory [69], had no history of neurological or psychiatric disorder, had no history of alcohol or drug abuse, were non-smoking, had no excessive alcohol or caffeine consumption, were not currently taking any medication significantly affecting the central nervous system, had no implanted metal, had no sensitivity or allergy to latex, had good or corrected hearing and vision, and reported no sleep disturbances. Women who were pregnant, or thought they may be, were also excluded. Forty-seven participants were recruited and forty-five signed informed consent. Four dropped out due to scheduling issues, and four dropped prematurely, two because they could not become comfortable enough to fall asleep in the laboratory, and two because they could not tolerate a different type of stimulation used in a different, concurrent study. A total of 37 participants (mean age = 21.34, SD = 3.47, 20 female) was included in the analysis. All participants provided signed informed consent to participate in the study in accordance with the Declaration of Helsinki, which was approved by the Chesapeake Institutional Review Board.

### 2.2. Procedures

**Figure 1:**
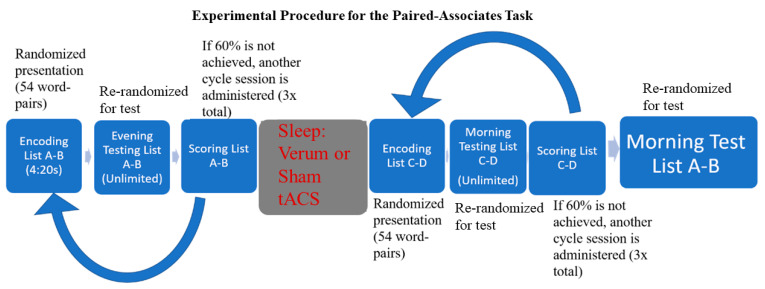
Experimental timeline for retroactive interference paired-associates task (ri-PAT) paradigm.

This study used a between-subjects design and occurred during the participants’ first (acclimation) night/morning in the sleep laboratory. Participants were randomly assigned to one of two conditions (verum or sham overnight stimulation). They arrived at the sleep lab at approximately 17:00 and list A-B learning began at approximately 21:00 p.m. Bedtime was set for 22:00 p.m, thus the retroactive interference PAT (ri-PAT) was the final task performed before sleep. Participants were seated in front of the testing computer approximately 24 inches from the screen. After studying list A-B, participants were administered the first list A-B test. If they did not accurately recall at least 60% of the word pairs [70], another encoding round was administered, followed by another test. This sequence was repeated up to three times or stopped once participants reached at least 60% accuracy. They were not given feedback regarding the word pairs that were correct or incorrect, but rather had to keep in mind the word pairs that may have been inaccurate and adjust their responses. They were then allowed to go to sleep. Prior to sleep, EEG electrode locations were digitized, and bio-calibrations were performed. Lights out for the participants occurred between 22:00–23:00, and they were allowed to sleep for eight uninterrupted hours before being woken up. During sleep, EEG data were monitored, and the closed-loop stimulation intervention was started when four minutes of continuous N2/N3 sleep was observed and allowed to run through the remainder of the night. Upon waking, participants could use the restroom and were offered water and snacks. Following the sleep session, participants were required to learn list C-D to at least 60% accuracy in the morning prior to taking the final test for list A-B. Participants left the sleep lab at approximately 08:00 following all testing (please see Figure 1 for the experimental timeline).

### 2.3. Tasks/Materials

Participants completed a verbal ri-PAT paradigm on a computer using custom-built software in Matlab Version 9.3 (2017b) [71]. The design was modeled after [6,47]. The task consisted of an encoding phase, where participants were given five seconds to study each word pair, and a testing phase where participants were given one of the words for each pair and had to produce the corresponding word. Criteria for learning the lists was set at 60% or greater [72]. Please see Supplementary Notes 2 from [47] for word lists. Participants responded by typing words into a text box. There was no time limit imposed on participants to produce a response.

### 2.4. Polysomnographic (PSG) Data Collection/Closed-Loop Transcranial Alternating Current Stimulation (CL-tACS) during Slow-Wave Oscillations

**Figure 2:**
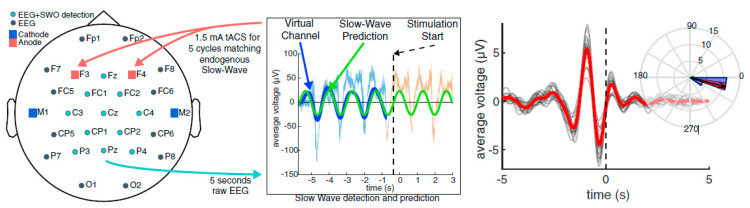
Left—Stimulation montage for closed-loop transcranial alternating current stimulation (CL-tACS) during slow-wave sleep (SWS). Middle/Right—CL-tACS procedure. The virtual electroencephalography (EEG) channel in the 5 s buffer is bandpass filtered in the slow-wave oscillation (SWO) frequency range (0.5–1.2 Hz). If the relative power in the SWO band is >20% of the broadband power across 0.1–250 Hz, a sine wave at the dominant SWO frequency is fit to the filtered virtual channel and projected into the future to predict the time points of the next available UP states. By matching the phase of the tACS to this projected function, the dynamics of tACS and the predicted endogenous signal are aligned. For verum stimulation, 5 cycles of CL-tACS were applied in response to observed SWO events through the sleep. Figure copied from [58] with permission.

For polysomnographic (PSG) data collection during sleep, a custom-built, 64-channel Neuroelectrics StarStim was utilized (Neuroelectrics, Inc., Barcelona, Spain). The closed-loop tACS algorithm measured ongoing EEG activity, calculating the presence of SWA, until a point where the ratio of SWA to broad-band activity reached a threshold. Once the threshold was reached, the algorithm triggered tACS to occur for 5 cycles at the endogenous phase and frequency of the ongoing SWA. These 5 cycle bursts were targeted at the SWO up-state (Figure 2). For the verum group, 1.5 mA/hemisphere stimulation was applied bilaterally at electrodes F5/6 and PO7/8. For the sham group, the setup was identical, but no stimulation was applied at predicted up states. For additional details about the recording procedure and closed loop stimulation, see [58].

### 2.5. Statistical Analysis

Learning was calculated as the difference between performance at the morning test and criteria performance from the last evening test. Words had to be exactly recalled and produced to be counted as correct. Stimulation count was used as a covariate, as previous work has shown evidence for a relationship between stimulation count and performance [57]. Data were analyzed in a Univariate Analysis of Covariance (ANCOVA) framework. The dependent variable was the difference in number of words recalled on list A-B overnight. For the low vs. high encoder variable, a median split was performed on participant performance to the first recall test following encoding. Participants who scored above the median were classified as high encoders, and participants who scored below the median were classified as low encoders. The difference in number of words recalled between the morning A-B test and the last A-B test before sleep was entered into a univariate ANCOVA. Stimulation count was entered as a covariate. Condition (2 levels—verum, sham) and encoding strength (2 levels—low, high) were entered as between-subjects variables. All alpha values were Bonferroni corrected for multiple comparisons. Data were analyzed with SPSS version 24 [73]. There were no significant differences between verum and sham nights in terms of the number of awakenings as determined by an experienced rater, suggesting that our CL-tACS intervention did not disturb sleep.

## 3. Results

### 3.1. Effects of CL-tACS and Interactions with Encoding Strength on Learning

**Figure 3:**
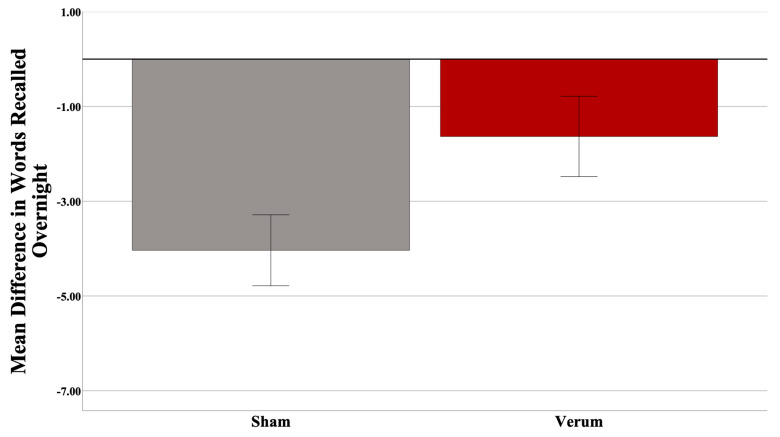
Significant behavioral effect (*p* = 0.04297) for verum tACS overall. Error bars = +/− 1 SEM.

**Figure 4:**
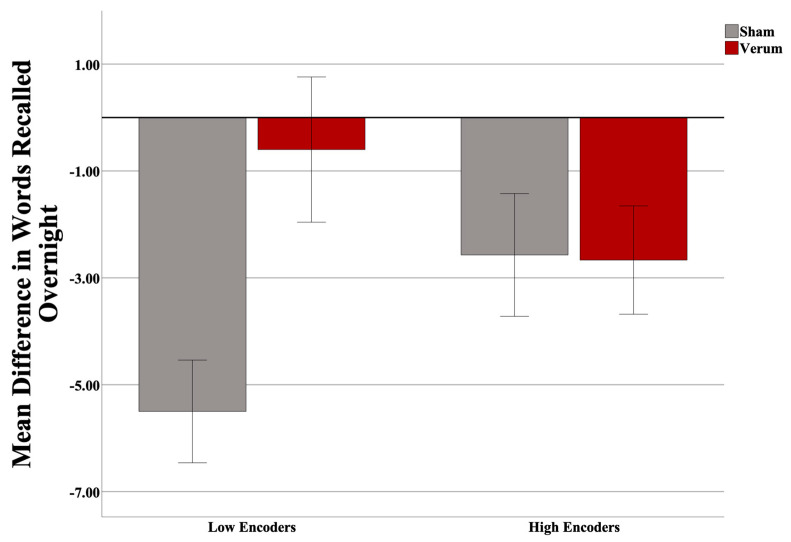
Interaction of encoding strength with stimulation condition. In low encoders, verum tACS led to more words being recalled than sham (*p* = 0.0066). Error bars = +/− 1 SEM.

Three participants were excluded (two low encoders and one high encoder) from the analysis upon inspection of the dependent variable (>3 SD from the mean), leaving a total of 31 participants (17 sham, 14 verum; 15 low encoders, 16 high encoders). At the final list A-B test in the evening (used to determine encoding performance), the high encoders on average recalled 10.6 more words than did the low encoders (out of a total 54 possible). There was no significant difference in Shipley IQ (*t*_(25)_ = −2.020, *p* = 0.054), nor Shipley Vocabulary Subscale (*t*_(27)_ = −1.378, *p* = 0.1794) between encoding groups, nor was there any difference in the number of attempts to reach criteria between groups (*p* > 0.05). The results suggested no effect of stimulation count on performance (*F_(1,26)_* = 0.531, *p* = 0.4727), and thus this covariate was taken out of the model. Gender was also investigated as predictor; however, it was not predictive overall, nor did it interact with other predictors, and thus was removed from the model. An independent samples *t*-test revealed no difference in predicted up states between verum and sham stimulation groups (*t_(25)_* = −0.832, *p* = 0.4134). A Univariate Analysis of Variance (ANOVA) was then run with an identical design without stimulation count as a covariate. A power analysis (G*Power version 3.1; [74]) revealed an achieved power of 0.759. Based on the effect size observed, 48 participants would have been required to have a study power of 0.80. Results suggest a significant overall effect of the stimulation condition (*F_(1,27)_* = 4.511, *p* = 0.04297; partial η^2^ = 0.143; see Figure 3), and a significant interaction of the encoding strength and stimulation condition (*F_(1,27)_* = 4.876, *p* = 0.03591; partial η^2^ = 0.153). Planned simple effects tests of stimulation condition within levels of encoding strength revealed a significant effect of stimulation for low (*F_(1,27)_* = 8.664, *p* = 0.0066; Cohen’s *d* = 1.075), but not high (*F_(1,27)_* = 0.004, *p* = 0.9509) encoders. Within low encoders, verum stimulation led to an increase in 4.90 in words recalled on average compared to sham. Within high encoders, only a negligible difference (0.95 average word difference) was observed. Please see Figure 4.

### 3.2. Effects of CL-tACS on Interference

**Figure 5:**
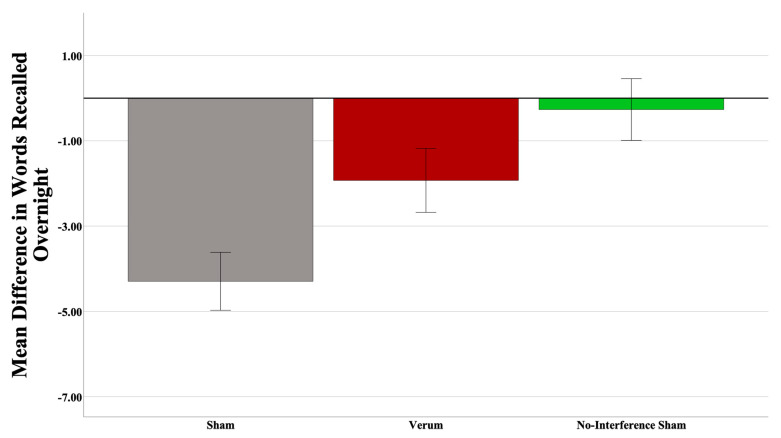
Interference is reduced with CL-tACS. A significant difference between the current experiment and PAT sham groups was observed, where the PAT sham group recalled significantly more words than did the current experiment sham group (*p* = 0.000613). There was no significant difference between the verum and PAT sham groups, which suggests CL-tACS does provide some protection from retroactive interference. Error bars = +/− 1 SEM.

To test the hypothesis that verum CL-tACS protects from retroactive interference, the difference in number of words recalled between the morning A-B test and the last A-B test before sleep was entered into a univariate ANOVA. Since the current experimental design did not have a condition where participants did not experience an interference (C-D) list in the morning prior to final A-B testing, the sham participants from a different, unpublished experiment called “PAT”, using a standard PAT paradigm (i.e., no interference list) with the same A-B list, were used as a non-interference control group. The experimental setup (e.g., EEG cap, procedures, etc.) and stimulation parameters in that experiment were identical to the current experiment. Given that the design of the PAT experiment did not have an interference component, the sham group from that experiment is a reasonable choice to answer the current hypothesis. Condition (3 levels—verum, sham, PAT sham) was entered as a between-subjects variable. One outlier (>SD from the mean) participant from the PAT sham group was removed prior to analysis. A power analysis revealed an achieved power of 0.964. The results suggested a main effect of the condition (*F_(1,43)_* = 8.378, *p* = 0.000846; partial η^2^ = 0.280). Pairwise comparisons within the condition revealed significant differences between the current experiment and the PAT sham groups (mean difference = 4.027, *p* = 0.000613; Cohen’s *d* = 1.438), where the PAT sham group recalled significantly more words than the current experiment sham group. Interestingly, there was no significant difference between the verum and the PAT sham groups (mean difference = 1.662, *p* = 0.353067), which suggests that CL-tACS does provide some protection from retroactive interference. Though not significant, the numerical difference between the verum group and the PAT sham group also suggests that there was an overall retroactive interference effect in the current study. Please see Figure 5.

## 4. Discussion

In this experiment, we aimed to investigate whether CL-tACS delivered during sleep can reduce retroactive interference from learning new information upon wake. A non-associative paired associates AB-CD interference paradigm was utilized. For this ri-PAT paradigm, we demonstrate that verum stimulation during sleep reduces retroactive interference compared to the sham. We first demonstrated that participants in the verum condition forgot on average 2.40 fewer words than sham overall, an effect on par with previous PAT studies. For example, [47] demonstrated a 2.69-word difference, with verum stimulation outperforming sham. However, within low encoding subjects, we showed a 4.90-word difference when comparing verum to sham, an effect twice the size of [47], although participants were not split into such categories in that study. Others have found similar effects in working memory performance [75,76,77]. The sham group from the no-interference PAT experiment was used to compare to the current groups. The PAT sham group forgot the least amount overnight, the verum group from the current experiment had intermediate performance, not significantly different from the PAT sham group, and the current experiment sham group forgot significantly more words than did the verum group in the current experiment and the sham group from the PAT experiment. There is evidence from the literature arguing that encoding strength as well as sleep interact to influence interference effects. Thus, perhaps these results show that our CL-tACS intervention is helping poor encoders compensate. The algorithm deployed here delivered tACS at the endogenous phase and frequency of brain activity. We propose that closed-loop tACS enhances the power of SWOs through the night, which boosts the transfer and consolidation of recently acquired task information, as well as reduced interference effects from learning new information in the morning following sleep on pre-sleep learning.

This study has focused on declarative memory consolidation, in the context of verbal paired associates learning. Future studies utilizing closed-loop tACS could be conducted with a more traditional assay of declarative memory consolidation. We used a PAT here, as it is a foundational task used in most of the previous electrically augmented sleep-dependent memory consolidation studies, which have delivered stimulation in an open-loop manner only. The learning criterion was set to 60% [72], however, some studies have participants learn the material to higher criteria, up to 100% [16]. Manipulating this criterion using the current stimulation paradigm could be an interesting way to elucidate the effect we observed on low vs. high encoders.

Our CL-tACS intervention can be optimized by personalizing the most critical parameter of the SWO relative power threshold using prior sleep data from a given participant. The participant’s whole-night polysomnographic recordings can be staged by an expert rater, to extract the distribution of relative power of the SWO band in the identified NREM sleep stage three (N3), when SWOs are most likely to occur [9]. Several studies show an improvement in learning with an intervention over the course of a nap (e.g., 60, 62), and, perhaps, our intervention would be more effective if we restricted its delivery to the first 3 h of sleep, when SWOs are the richest.

### 4.1. Limitations

Sleep research presents a host of unique problems with data collection, including participant comfort. One limitation of electrical stimulation during sleep, as in wake, is that a small subset of participants cannot tolerate the physical sensations associated with brain stimulation. Thus, it could be argued that electrical stimulation is suboptimal to auditory stimulation. However, the incidence of intolerability for electrical stimulation is exceptionally low on average, and electrical stimulation is robust against interference from ambient sensory stimuli in the environment owing to its non-sensory nature.

Given the findings in the literature, including this work, a careful study should be undertaken that investigates just these tasks with our closed-loop tACS intervention. These experiments were part of a larger study, and there were likely confounds that could have influenced the results reported here in the form of interference, sleepiness, etc. For example, the PAT experiment and the current experiment were conducted without an adaptation night, which is standard practice in sleep studies. There is a known “first-night effect”, where participants’ sleep is disrupted the first night sleeping in the laboratory [78]. Data regarding participant sleep quality, measured via actigraphy or sleep diary, was not collected for this study, nor were any daily activity/work data collected. Participants were excluded from the study if they reported a non-normal sleep schedule (due to late-shift work, parasomnia, etc.). The results here are encouraging and should be validated with a carefully controlled replication study. A within-subjects design would be desirable to control individual differences in encoding strength, sleep architecture, memory capacity, EEG dynamics, etc. A more comprehensive assessment of participant coding ability was not employed here; rather, performance from one measure was used to characterize encoding ability. Finally, no investigation of the neurophysiological effects (i.e., slow wave EEG power) during the night following stimulation was performed for this study. Though this was shown in previous work with the same hardware and software to modulate slow-wave power [58].

### 4.2. Future Directions

Future work will include an item-by-item analysis for the ri-PAT paradigm. In the current results, weak encoders showed more improvement, which could be because weakly encoded memories are preferentially reactivated or because the memory isolation hypothesis shelters them from being activated/interfered with, but the best way to test that would be to either copy a previous design (where words are studied multiple times or up to a specific criteria) to test, or by doing an item (correctly encoded/not) by item analysis of these data.

To talk about a phenomenon mechanistically, it is necessary that selective enhancement or impairment be accomplished. Closed-loop stimulation methods have the capability to both enhance (by stimulating in phase) and disrupt (by stimulating out of phase, via a different frequency, or location; see [79]) endogenous brain rhythms, thus, theoretically, it would be possible to improve the consolidation of some information while impairing the consolidation of other information. More studies should be conducted that selectively impair consolidation, including the task presented here. The idea of targeted memory consolidation (TMC) could allow for the possibility of selectively augmenting memories and deciding externally which ones endure and which are driven to perish. This could be beneficial to students, those stricken with phobias, post-traumatic stress disorder (PTSD), or addiction, by selectively targeting painful or maladaptive memories for reactivation and subsequent disruption of reconsolidation.

Memory consolidation can be disrupted via tACS. In [71], cross-hemispheric tACS during SWS was applied in an afternoon nap, with methods such as those outlined in [47], disrupting both slow wave activity and memory consolidation. Eight subjects participated in a cross-over within subject design where two naps were taken, during which either active or sham stimulation was applied during SWS. Declarative memory was assessed with a PAT. Though non-significant, this stimulation procedure produced a decrement in recall following the nap, and this decrement was associated with a significant reduction in slow oscillations compared with sham stimulation. Interestingly, SWA showed a rebound, reflecting homeostatic sleep pressure, following the final stimulation interval compared to the sham.

Impairing memory consolidation is an important potential aspect of understanding the underlying mechanisms of memory in sleep. The ability to selectively activate traumatic memories, for example, then disrupt the reconsolidation procedure could be a valuable treatment protocol for phobias, anxiety disorders, PTSD, addiction, or other disorders. In [80], two studies were reviewed, one in mice and one in humans, showing that manipulating sleep can lead to the forgetting of fearful associations. In the first study, mice were conditioned to fear an odor that was paired with a foot-shock. The odor was then presented during SWS, and an injection of an amygdala protein-synthesis inhibitor was delivered. This intervention led to a diminution of fear expression when tested 24 h later. Interestingly in humans, a conditioned fear response to faces was diminished after odors that were associated with the conditioned fear were presented during SWS, without the need of a protein-synthesis inhibitor. The conditioned fear response decreased throughout the sleep period, indicating that TMR during sleep was reorganizing the memory from a fearful to a safe one. These changes were also associated with decreased hippocampal activation and the reorganization of ensemble pattern activity in the amygdala, as revealed with neuroimaging.

## 5. Conclusions

In conclusion, this series of experiments sought to investigate the effects of closed-loop tACS on memory consolidation in a canonical task (paired associates), but also by expanding the scope of tasks and theoretical constructs (retroactive interference) to further elucidate the possibilities of stimulation during sleep to improve memory consolidation. We show here that closed-loop tACS protects from retroactive interference in a PAT, specifically for those on the lower half of the encoding spectrum (low-encoders). This work adds to a growing body of research from our laboratory suggesting modulation of the brain via tES improves performance in a variety of tasks, including visual target detection [57,58,81,82,83,84,85], working memory [86,87], episodic metamemory [44,45], and insight into temporal rules [88]. These results are encouraging, but future work is needed to better understand the underlying brain mechanisms of stimulation-induced sleep-dependent memory consolidation enhancement and protection from retroactive interference.

## 6. Patents

J. Choe and P.K. Pilly (2019). Method for low latency automated closed-loop synchronization of neurostimulation interventions to neurophysiological activity. US Patent No. 10,413,724.

## Data Availability

Data supporting reported results may be obtained through consultation with the study authors.
